# Circular RNA SMO sponges miR-338-3p to promote the growth of glioma by enhancing the expression of SMO

**DOI:** 10.18632/aging.102576

**Published:** 2019-12-30

**Authors:** Zhiyong Xiong, Chaoyang Zhou, Luyang Wang, Ronglan Zhu, Liangchen Zhong, Dengfeng Wan, Qiangping Wang

**Affiliations:** 1 Department of Neurosurgery, Union Hospital, Tongji Medical College, Huazhong University of Science and Technology, Wuhan 430022, China; 2 Department of Neurosurgery, Jiangxi Provincial People's Hospital Affiliated to Nanchang University, Nanchang 330006, China; 3 Department of Neurology, The Central Hospital of Wuhan, Tongji Medical College, Huazhong University of Science and Technology, Wuhan 430014, China

**Keywords:** glioma, circRNA, circSMO742, miR-338-3p, SMO

## Abstract

Glioma is one of the most common tumors in the brain and complete cure still a challenge. The present research aimed to investigate the molecular mechanism of circular RNA SMO (circSMO742) in glioma, via targeting miR-338-3p and regulating SMO expression. QRT-PCR was utilized to examine the expression profiles of circSMO742 and microRNA-338-3p (miR-338-3p) in glioma. SMO protein in glioma was tested via western blot. RNA pulldown assay and dual luciferase reporter assays were used to explore the targeting correlation between RNAs. MTT assay, transwell assays and flow cytometry were used to investigate cell proliferation, migration and invasion, and apoptosis, respectively. Tumor xenograft was done to ascertain the effect of circSMO742 knocking down on tumor growth. CircSMO742 and SMO were highly expressed in glioma tissues, while miR-338-3p expression was reduced. CircSMO742 together with SMO could promote cells proliferation, migration and invasion while inhibit cells apoptosis, whereas miR-338-3p showed negative impacts on the cell activity. Knocking down of circSMO742 suppressed glioma growing *in vivo*. CircSMO742 promoted glioma growth by sponging miR-338-3p to regulate SMO expression. Our research revealed a new molecular mechanism of glioma growth and provide a fresh perspective on circRNAs in glioma progression.

## INTRODUCTION

As one of the most common type of tumors in brain, glioma accounts for 60% of central nervous system tumors, and 80% of all malignant brain tumors [1]. Glioma is prevalent, with the highest incidence of intracranial tumors [2]. The median survival period for glioblastomas patients is 12 to 15 months, and that for anaplastic gliomas patients is 2–5 years [3]. Patients often suffer from vomiting, headaches, nerve distortion, sensory deficits, and bear the pain of recurring, resulting in low life quality [4]. Although standard emerging methods like imaging, surgery, radiotherapy have been adopted to remove the glioma, it still remains incurable [5, 6]. Therefore, an in-depth understanding of the genetic mechanism towards glioma is significant for its prevention, diagnosis, and treatment.

Circular RNAs (circRNAs), a kind of non-protein-coding RNAs, are formed by a specific back splicing from pre-mRNA or a ligation of 5′ and 3′ ends of linear RNAs [7]. CircRNAs was firstly found in 1964 and have been widely studied in the recent years, so more and more circRNAs were found to have correlation with gene regulation and related to various diseases [8]. For example, Bing Chen et al demonstrated that circRNAs could serve as cancer regulator and biomarker since they suppressed or promoted cancer in different cancers, such as adenocarcinomas, bladder carcinoma, hepatocellular carcinoma and glioma [9]. To be specific, circTTBK2 could was revealed to promote glioma proliferation by regulating oncogenic factor HNF1β via binding to miR-217/Derlin-1 pathway [10]. Yang P. et al found that glioma tube growth could be suppressed with by circ-ZNF292 silencing, which can limit cell cycle progression at S/G2/M stage through the Wnt/β-catenin signaling pathway and gene regulations like PRR11, Cyclin A, p-CDK2, VEGFR-1/2, p-VEGFR-1/2 and EGFR [11]. In spite of that there are a few researches towards glioma-related circRNAs, the related evidence is still limited.

MicroRNAs (miRNAs), another family of non-coding RNAs with the length of 20-24 nucleotide, were reported to be a star target involved in tumorigenesis, angiogenesis, and drug resistance. The miRNAs can regulate gene expression by binding to various mRNA as a post-transcriptional or translational level [12, 13]. There are abundant evidence suggesting that miRNAs play a crucial role in glioma. For instance, Nie S. et al demonstrated that miR-495 induced the change of metabolism in glioma cells by increasingly Glut1 expression, resulting in the increase of glucose-taking and glioma developing [14]. MiRNA-221 and miRNA-222 were found to be highly expressed in glioma and the expression of which was closely related to survival rates of glioma cells [15]. Herein we provided an insight into the relationship of glioma and another miRNA, miR-338-3p, which have been discovered essential in the development of non-small-cell lung carcinoma, ovarian epithelial carcinoma, and gastric cancer [16–18].

Smoothened (SMO), a 7-transmembrane G protein-coupled receptor, plays a significant role in regulating Hedgehog signaling pathway [19]. SMO regulated genes expression by transducing GLI1 from the cell’s cytoplasm to the nucleus, which increases cell cycle progression and inhibits apoptosis [20]. It was also reported that SMO enhanced cells migration and proliferation and was activated in caner tissues than in normal one [21]. For example, Ding Y. and his colleagues revealed that SMO was highly expressed in colon cancer, which could be a potential biomarker of colon cancer [22]. The Same changes could be found in basal cell carcinomas, leukemia and glioma [20, 23]. However, the regulation mechanism of SMO in glioma is still not clear.

Here, we explored the mechanism that how circSMO742 affected glioma. We researched expression profiles of circSMO742 as well as its effect on cells proliferation, apoptosis, invasion and migration. Further, we investigated the specific miRNA, miR-338-3p, that circSMO742 targeted and the interaction among circSMO742, miR-338-3p and SMO. Besides, their function and interaction in the process of tumor formation were also explored through tumor xenograft assays in nude mice. These researches revealed a possible pathogenesis mechanism of glioma and suggested a potential therapeutic target.

## RESULTS

### Analysis of endogenous competition mechanism relationship according to the microarray chip

**Figure 1:**
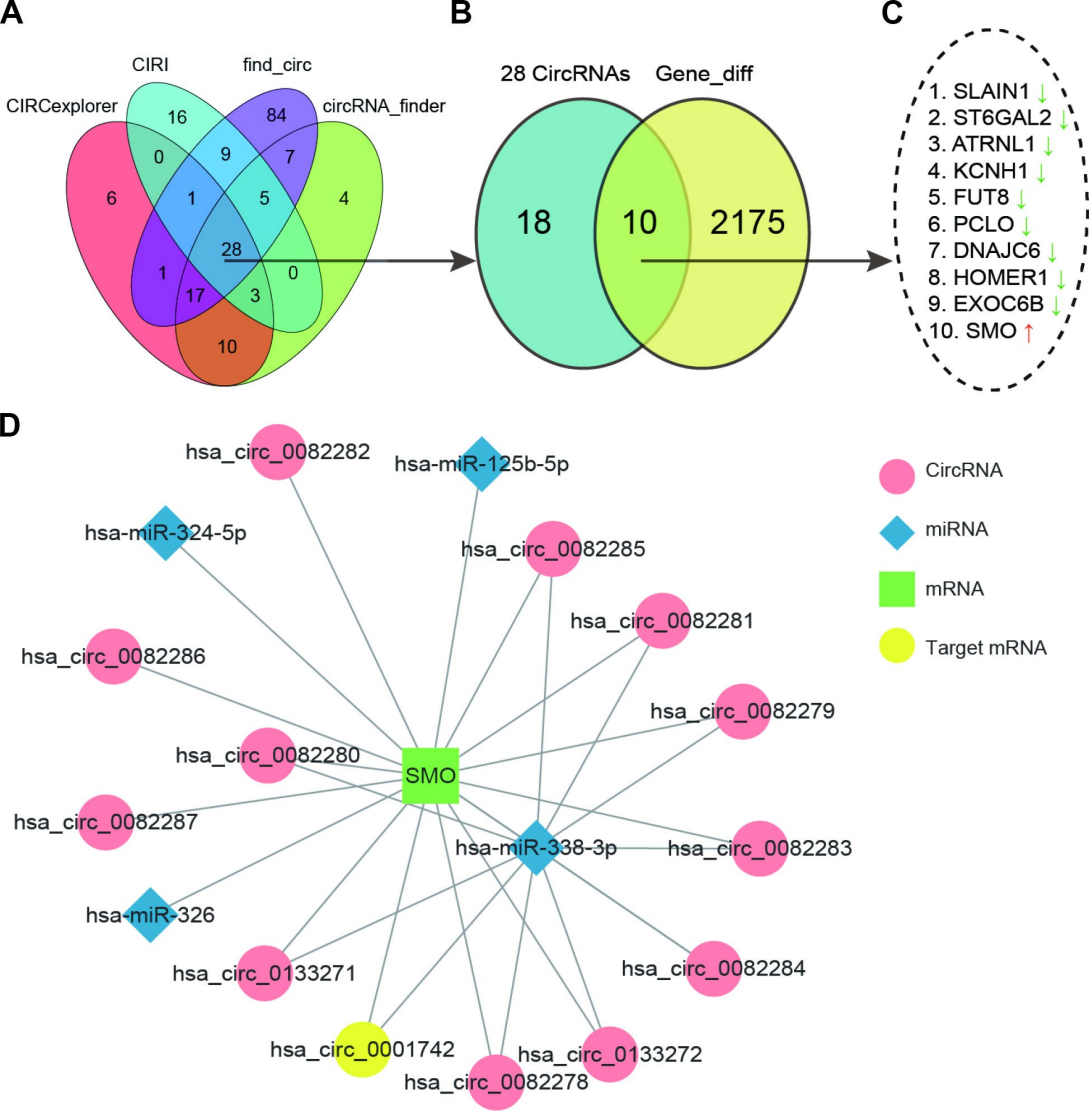
**CircSMO742 was predicted as an important factor in glioma.** (**A**) 28 common differently expressed circRNAs in GSE86202 searched with four methods shown by Venn diagram. (**B**, **C**) 10 DEGs sharing same name by these 28 circRNA and differently expressed mRNAs with RNA-Seq, and only SMO up regulated. (**D**) Network of mRNAs transcripted by SMO, circRNAs and possible targeting miRNAs.

We screened out differently expressed circRNAs from GSE86202 (6 samples, including 3 gliomas samples and 3 paired normal brain tissue) by using CIRCexplorer, CIRI, find_circ and circRNA_finder respectively. Than we got 28 common circRNAs were got by diffuse analysis with limma, shown in Table 1 (Figure 1A). Ten differentially expressed genes (DEGs) was shared the same name with these 28 circRNAs. Searching ten differently expressed mRNAs with RNA-Seq (Figure 1B) and Only SMO was up-regulated among the ten DEGs while other genes were all down-regulated (Figure 1C). We further investigated the network of mRNA which transcripted by SMO, including circRNAs and possible targeting miRNAs. We found SMO could target many circRNAs but only hsa_circ_00001742 (circSMO742) was differently expressed in glioma. Moreover, SMO and circSMO742 all had target relationship with miR-338-3p (Figure 1D). Thus, we speculated that SMO could competitively bind to circSMO742 with miR-338-3p in glioma.

**Table 1:** 28 common elements in "CIRCexplorer_diff", "circRNA_finder_diff", "CIRI_diff" and "find_circ_diff".

**CircBase ID**	**CircRNA FD**	**Adjusted p Value**	**Gene name**	**Gene expression**
hsa_circ_0000497	-5.6221	0.0048	SLAIN1	down
hsa_circ_0078784	-5.3571	0.0085	PSMB1	-
hsa_circ_0001368	-4.8097	0.0105	KLHL24	-
hsa_circ_0055954	-4.7531	0.0197	ST6GAL2	down
hsa_circ_0079422	-4.6018	0.0287	ICA1	-
hsa_circ_0064555	-4.4995	0.0267	SATB1	-
hsa_circ_0020094	-4.4333	0.0308	ATRNL1	down
hsa_circ_0006410	-4.4025	0.0305	TUSC3	-
hsa_circ_0004238	-4.3673	0.0308	PTK2	-
hsa_circ_0016351	-4.3629	0.0339	KCNH1	down
hsa_circ_0002904	-4.3505	0.0267	LPXN	-
hsa_circ_0000836	-4.3279	0.0309	MIB1	-
hsa_circ_0064615	-4.2387	0.0362	SLC4A7	-
hsa_circ_0003028	-4.0358	0.0155	FUT8	down
hsa_circ_0080941	-3.9832	0.0485	PCLO	down
hsa_circ_0024997	-3.9648	0.0362	ERC1	-
hsa_circ_0005114	-3.8978	0.0213	RIMS2	-
hsa_circ_0004425	-3.0923	0.0339	TMEFF1	-
hsa_circ_0002454	-2.7503	0.0085	DNAJC6	down
hsa_circ_0006916	-2.4156	0.0085	HOMER1	down
hsa_circ_0002158	-2.3507	0.0085	RERE	-
hsa_circ_0009043	-2.2562	0.0157	EXOC6B	down
hsa_circ_0000118	-2.0537	0.0085	MAN1A2	-
hsa_circ_0007364	-1.9031	0.0305	PTP4A2	-
hsa_circ_0005660	3.3744	0.0308	NFIX	-
hsa_circ_0001742	3.5249	0.0105	SMO	up
hsa_circ_0001573	3.5875	0.0267	RREB1	-
hsa_circ_0008016	4.1072	0.0105	FGFR1	-

FD: foldchange value according to CIRCexplorer, negative means low expression, positive means high expression. “-” means no different expression.

### An opposite expression relationship of circSMO742 and miR-338-3p existed in glioma

**Figure 2:**
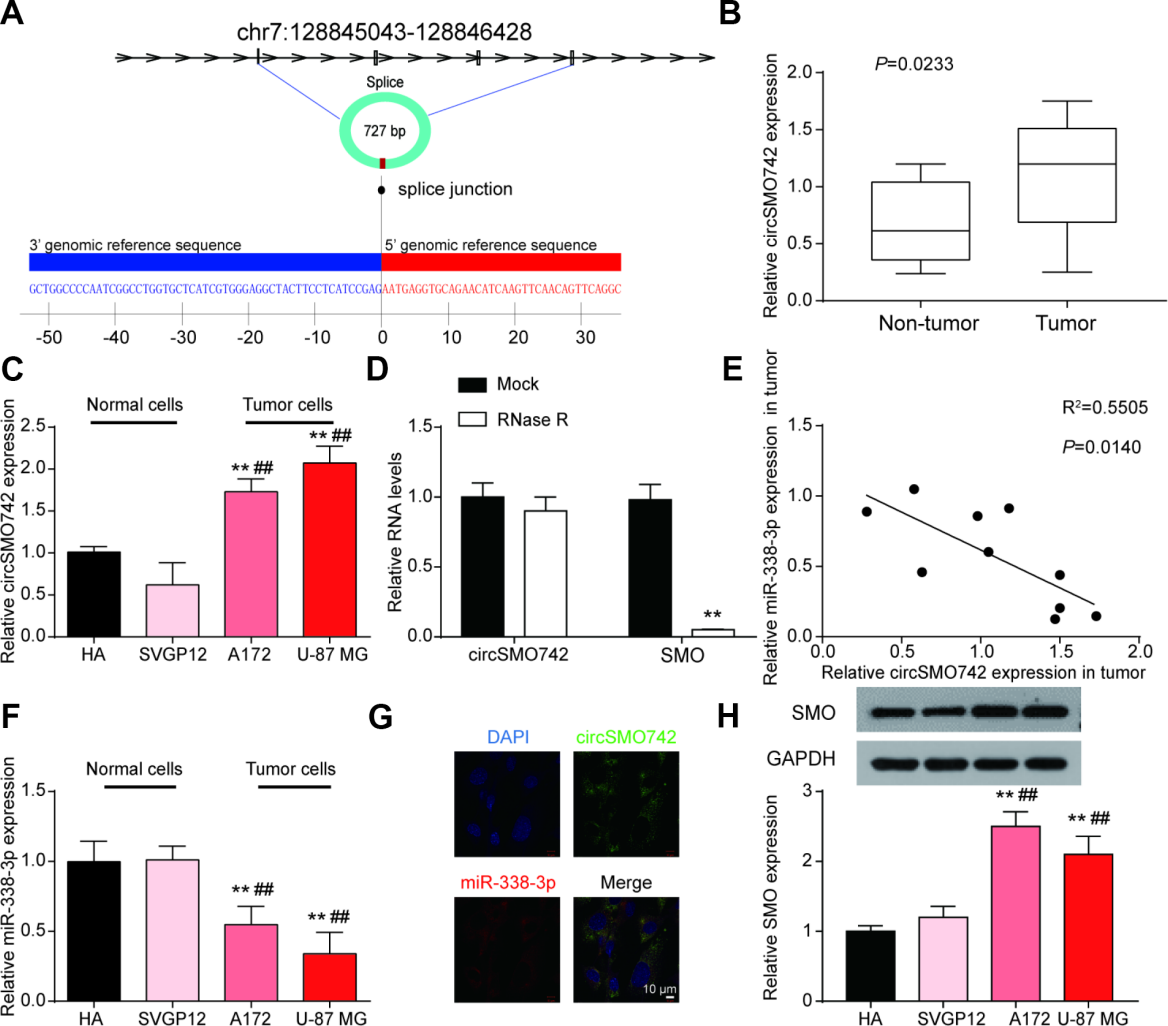
**CircSMO742 and miR-338-3p had an opposite expression in glioma.** (**A**) The mosaic site sequence information of circSMO742. (**B**) Higher expression level of circSMO742 in tumor tissue than in non-tumor tissues in 10 tumor samples, *P*<0.05. (**C**) Higher level of circSMO742 expression in A172 and U-87 MG, measured with qRT-PCR. (**D**) RNase eliminated the SMO mRNA. (**E**) Negative correlation of miR-338-3p and circSMO742 in 10 tumor tissues, R^2^=0.5505, *P*<0.05. (**F**) lower level of miR-338-3p expression in A172 and U-87 MG, measured with qRT-PCR. (**G**) CircSMO742 was located in the cytoplasm. (**H**) Lower expression level of SMO proteins in A172 and U-87 MG, detected with western blot assay. *P<0.05, **P<0.01, ***P<0.001, v.s. HA; #*P*<0.05, ##*P*<0.01, ###*P*<0.001, v.s. SVGP12.

To verify our hypothesis, we examined the expression profiles of circSMO742, miR-338-3p and SMO. CircSMO742 is cyclized with the four exons of chromosome 7 with a total length of 727 base pair. The mosaic site sequence information is shown in the Figure 2A. In comparison with non-tumor tissues, circSMO742 was highly expressed in 10 tumor samples (*P*<0.05, Figure 2B). The expression levels of circSMO742, miR-338-3p and SMO proteins were measured in HA, SVGP12, A172 and U-87 MG cells lines, respectively. As the results shown that circSMO742 and SMO protein had higher protein expression levels while miR-338-3p had lower expression level in tumor cells, compared with normal cells (Figure 2C, 2F, 2H). Spearman analysis indicated that the expression level of miR-338-3p was negatively related with circSMO742742 in 10 tumor tissues (Figure 2E). Moreover, circSMO742 was resistant to RNase R, indicating that circSMO742 is circular (Figure 2D). Fluorescence in situ hybridization (FISH) against circSMO742 showed circSMO742 was mainly distributed in cytoplasmic. (Figure 2G).

### Regulations among circSMO742, miR-338-3p and SMO

**Figure 3:**
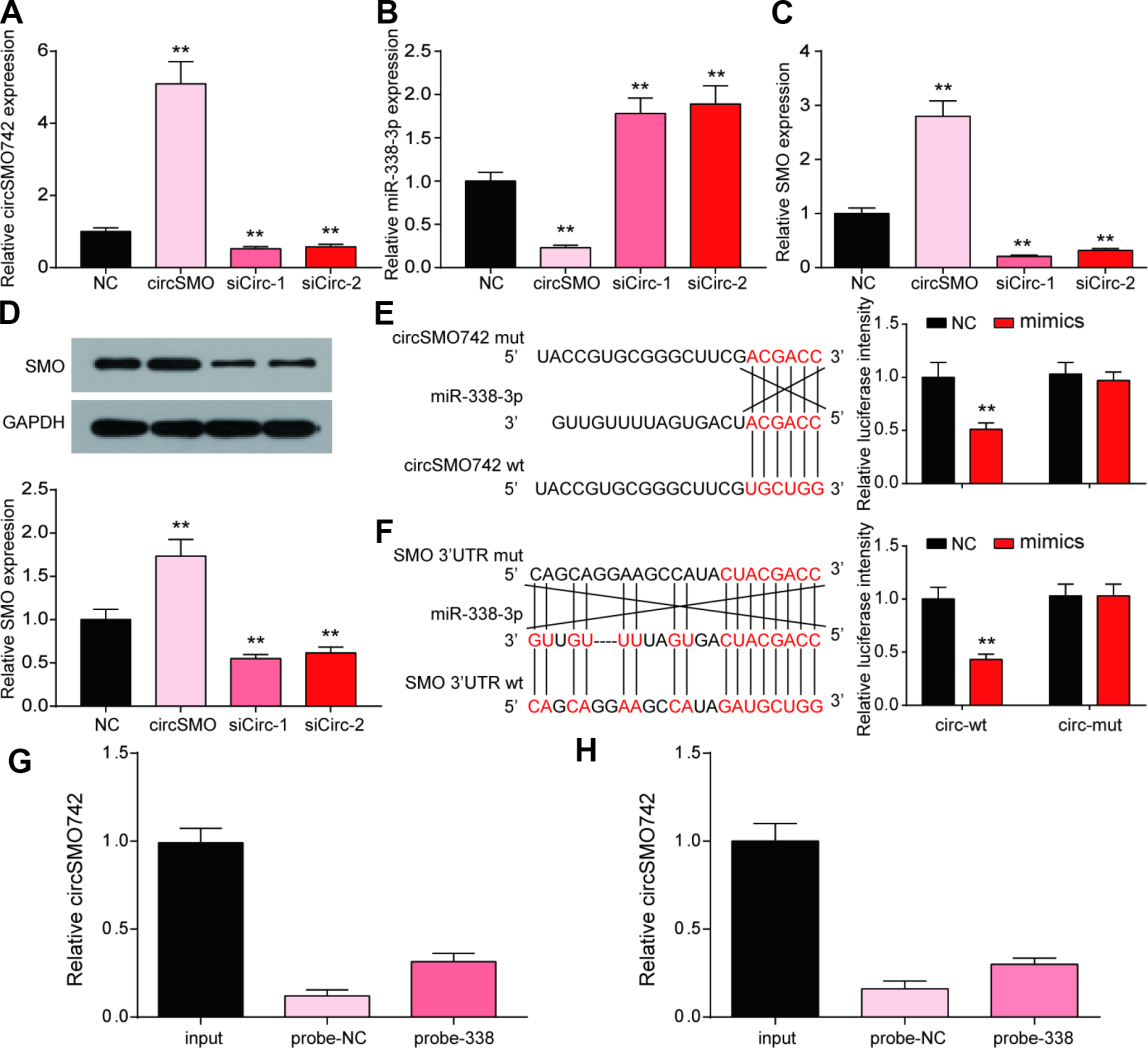
**CircSMO742 and SMO were simultaneously targeted by miR-338-3p.** (**A**–**D**) Expression level of circSMO742, miR-338-3p, SMO mRNA and protein in different groups, NC, circSMO742 over-expressed, circSMO742 knockdown, measured with qRT-PCR and western blot assay. The level of circSMO742 (**E**) and SMO (**G**) in U-87 MG cells captured with miR-338-3p-biotin probes were higher compared with NC-probe, respectively. The direct interaction between circSMO742 and miR-338-3p (**F**) or SMO and miR-338-3p (**H**) was detected by dual luciferase reporter assays. *P<0.05, v.s. NC group.

To clarify the interaction between circSMO742, miR-338-3p and SMO, transfection experiments in U-87 MG were performed with overexpressing circSMO742 and knocking down circSMO742 siRNAs respectively (Figure 3A). The results showed that overexpression of circSMO742 suppressed miR-338-3p while downregulation of circSMO742 enhanced it, and the SMO mRNA expression level showed same shift tendency as circSMO742 (Figure 3B, 3C). The result of western Blot was consistent to the transfection experiment (Figure 3D). The circSMO742 and SMO mRNA in U-87 MG cells can be captured with miR-338-3p-specific probes respectively. Similarly, dual luciferase reporter assays in U-87 MG cells demonstrated the existence of targeting relationship between circSMO742 and miR-338-3p (Figure 3E, 3F), as well as miR-338-3p and SMO mRNA (Figure 3G, 3H).

### CircSMO742 promotes the growth of glioma cells

**Figure 4:**
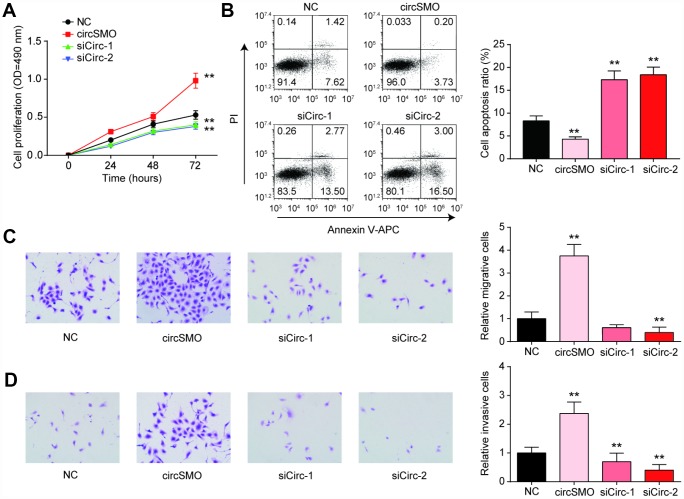
**CircSMO742 affected glioma cells activity.** (**A**) Cells proliferation in 72 hours measured with CCK8 assay, after over-expressed, or knocking down of circSMO742, compared with NC group. (**B**) Cells apoptosis ratio detected with Flow cytometry dealt with over-expressed, knocking down of circSMO742, compare with NC group. (**C**, **D**) Migration and invasion were detected with transwell assay after transfection. The results pointed out that circSMO promoted cells proliferation, migration and invasion while inhibited cells apoptosis. *P<0.05, compared with NC.

The cell proliferation of U-87 MG cells was enhanced with circSMO742 upregulation, while the siCircs had contrary effect (Figure 4A). Similarly, the same experiment was done in HA cell lines, while transfection of circSMO742 have no effect on cell proliferation (Supplementary Figure 1A). Moreover, cell apoptosis was reduced with circSMO742 overexpression and was increased when it was inhibited (Figure 4B). The results of transwell assay indicated that circSMO142 could promote cells mobility (Figure 4C, 4D).

### MiR-338-3p inhibited the growth of glioma cells

**Figure 5:**
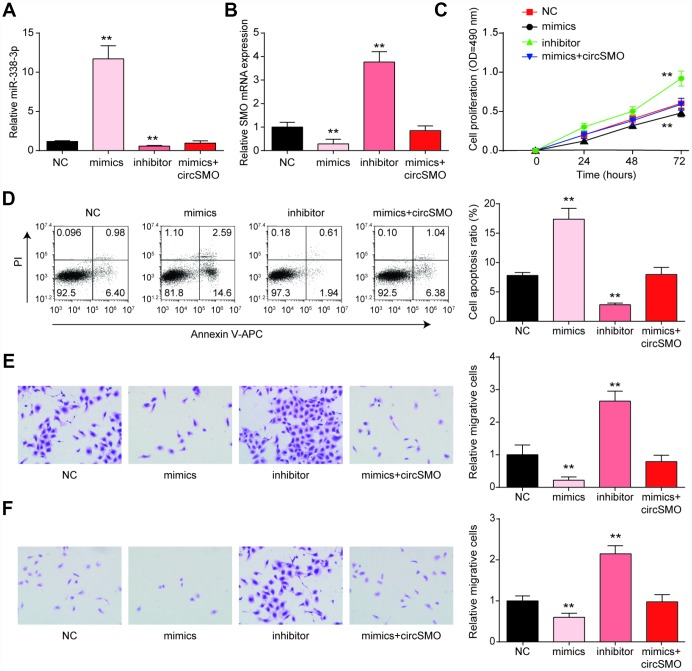
**MiR-338-3p inhibited the growth of glioma cells.** (**A**) miR-338-3p expression with different treating, miR-338-3p mimics and miR-338-3p inhibitor. (**B**) MiR-338-3p inhibitor promoted SMO mRNA while mimic inhibited it. (**C**–**F**) MiR-338-3p mimics inhibited cells proliferation, migration and invasion, while enhanced cells apoptosis according to assays of the CCK8, flow cytometry and Transwell. *P<0.05, compared with NC.

We continually researched how miR-338-3p affected tumor cells activity. The level of miR-338-3p expression with different treating were measured and the outcomes showed that miR-338p-3p level was higher with mimic treating whereas it was lower in the presence of inhibitor (Figure 5A). As for SMO mRNA, it was lower expressed with mimic treating whereas inhibitor could enhance the mRNA level. The circSMO742 could revert the role of mimic treatment (Figure 5B). When treated with miR-338-3p mimics, Cell proliferation of U-87 MG cells was decreased while in those treated by inhibitor was increased (Figure 5C). Besides, in the same experiment performed in HA cell lines, these four groups with different treatments displayed no significant differences on cell proliferation (Supplementary Figure 1B). Detection of cell apoptosis indicated that mimic treatment could promote cell death and this phenomenon could be reversed by circSMO742 overexpression (Figure 5D), miR-338-3p inhibited glioblastoma cells proliferation, migration and invasion in the CCK8 assay and transwell assay. However, this miRNA accelerated cells apoptosis as flow cytometry showed. However, it has no effect on HA cells (Figure 5E, 5F).

### CircSMO742 and miR-338-3p regulates SMO influencing the growth of glioma cells *in vitro* and *in vivo*

**Figure 6:**
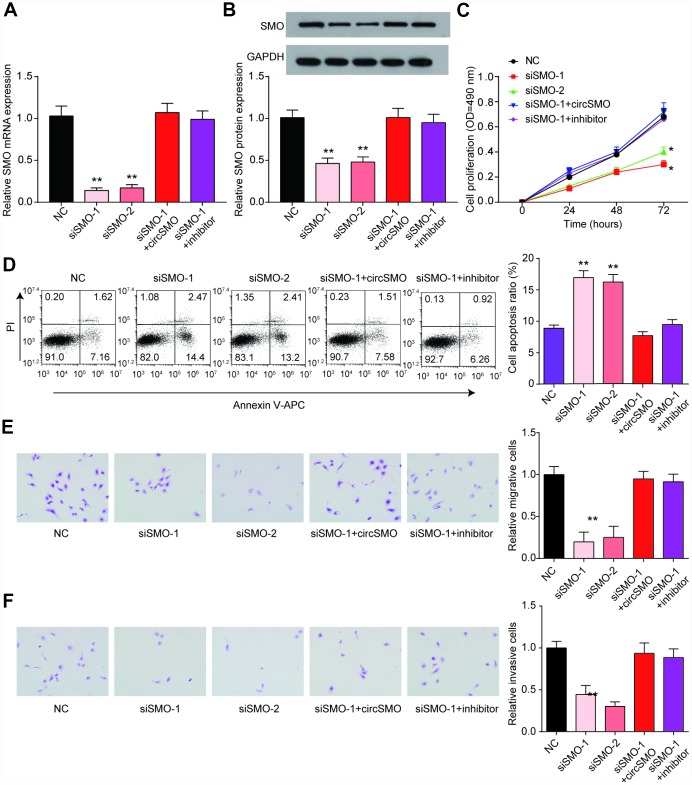
**SMO has same effect on cells activity as circSMO742.** (**A**, **B**) SMO knockdown inhibited SMO mRNA expression while the presence of circSMO742 or miR-338-3p inhibitor could reverse the expression caused by SMO knockdown. (**C**–**F**) SMO suppression suppressed proliferation, migration, invasion but promoted apoptosis of U-87 MG cells. *P<0.05, v.s. NC.

The SMO mRNA and protein levels were lowly expressed with SMO knockdown but could recover by circSMO742 or miR-338-3p inhibitor (Figure 6A, 6B). In U-87 MG cells, downregulation of SMO inhibited cells proliferation, migration, invasion and promoted cells apoptosis, (Figure 6C–6F) but had no remarkable influence on HA cell proliferation (Supplementary Figure 1C).

**Figure 7:**
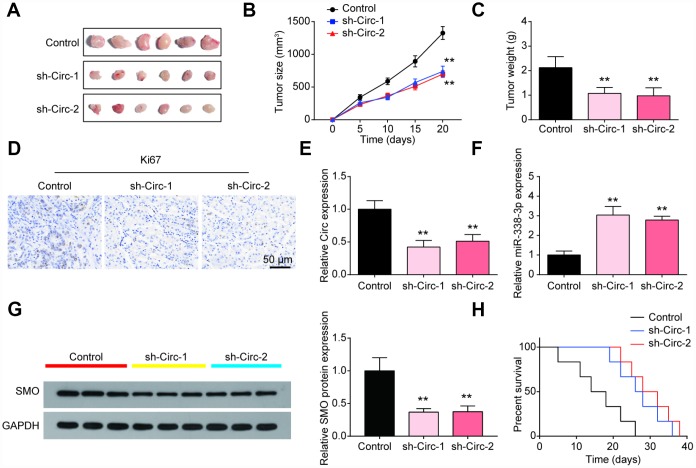
**The inhibitory role of circSMO742 knockdown was verified *in vivo*.** (**A**–**C**) The tumors in circSMO742 suppression group were significantly smaller than that of NC group after 20 days injections growth. (**D**) The Ki67 was stained to detect the proliferation of cells. (**E**–**G**) CircSMO742 and SMO protein expression in circSMO742 knockdown (sh-Circs) groups were reduced while miR-338-3p was elevated. (**H**) Survival percentages of nude mice with different treatments. **P<0.01, compared with control.

To further investigate the effect of circSMO742 on tumor growth rate, xenograft models were constructed with nude mice. As result shown (Figure 7A–7C), tumor formation in circSMO742 knockdown groups (sh-Circ-1& sh-Circ -2) was suppressed compared with the control group. Additionally, Ki67 staining showed lower cell proliferation level in circSMO742 knockdown groups, compared with NC group (Figure 7D). Western blot and qRT-PCR results showed that SMO proteins and mRNAs were reduced in circSMO742 knockdown groups but miR-338-3p was increased, compared to NC group (Figure 7E–7G). As shown in Figure 7H, intracranial tumor was planted, and the survival analysis indicate that circSMO742 knockdown groups exhibited longer survival time than the control group. These above results suggested that knocking down circSMO742 suppressed the growth of glioma via miR-338-3p and SMO, and improved the animal survival.

**Figure 8:**
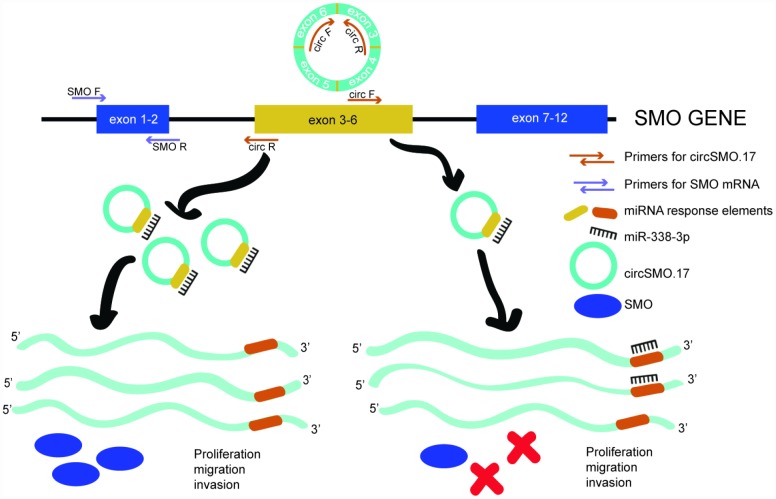
**Schematic diagram of endogenous competition mechanism.** CircSMO742 and SMO mRNA derived from a parent gene of SMO and circSMO742 circled from 4 exons. In glioma, circSMO742 was up-regulation could sponge miR-338-3p a lot to let parent gene largely coding SMO mRNA, encoding SMO protein to promote cell proliferation, migration and invasion.

Collectively, we may infer from these results that the abnormal expression of circSMO742 caused that only a little miR-338-3p could be free to target SMO mRNA in glioma, so SMO proteins level was higher to promote glioma growth through cells proliferation, migration and invasion. If we inhibited the circSMO742 expression through exogenous siRNAs transfection, miR-338-3p would increase and target SMO mRNA, so SMO proteins would be consequently reduced, and thus cell growth was inhibited (Figure 8).

## DISCUSSION

Our present study uncovered the molecular mechanism of circSMO742 in regulating glioma cells growth. Compared with normal cells, CircSMO742 was higher expressed in glioma cells, compared with normal cells, and could promote cells proliferation, migration and invasion while suppress cells apoptosis. MiR-338-3p was lowly expressed in glioma cells and had contrary influence on cells activity. SMO was found highly expressed and promote cells proliferation. CircSMO742 and miR-338-3p regulated SMO and affected the growth of glioma cells *in vitro* and *in vivo*. Knocking- down of circSMO suppressed the tumor growth and malignant progression significantly, and thus showed good potential in treating glioma.

In the past few years, circRNAs have been widely researched as an essential factor in regulating genes expression related to disease such as glioma. Such circRNAs was reported as a biomarker and showed great potential in disease treatment. For instance, inhibition of circ-TTBK2, functioned as miR-217 sponge, was found to restrain tumor proliferation via down regulating HNF1β and Derlin-1 [10]. It had reported Circ-FBXW7 was decreased in glioma tissues cells, and the overexpression of Circ-FBXW7 would help inhibit cancer cells cycle by regulating the encoding of a proteins, FBXW7-185aa [24]. Notwithstanding, the research towards circRNAs related to glioma is far from comprehensive and more exploration is needed. In this study, we suggested a novel circRNA, hsa_circ_00001742 (circSMO742), which never had been discussed as far as we know, in regulating gliomas.

According to the previous study, miR-338-3p had competitive relationship with the circSMO742 which transcripted by SMO, so we assumed that it might have targeting relationship with circSMO742 [25]. MiR-338-3p was reported to have correlation with tumor differentiation and down regulation of which could suppress terminal glial differentiation [26]. Also, miR-338-3p had been found to be down-regulated in glioma cells [27] and could inhibit malignant biological behaviors of glioma cells, which is consistent with our findings [28]. Yet, we focused on new targeting relationship between circSMO742 and miR-338-3p and the results enriched the regulation mechanism of that.

It has been reported that circRNA served as a miRNA sponge by binding with related miRNA sequences and this is considered as a general phenomenon existing in circRNA-miRNAs-mRNA axis [29]. As competitive miRNA binders, circRNAs displayed better capability to sponge miRNAs than mRNAs and subsequently strongly suppressed the activity of miRNAs to bind to its target mRNAs [30]. Therefore, we deemed that circSMO742 had specific sponging action for miR-338-3p, so the binding between miR-338-3p and SMO mRNA could be inhibited by the existence of circSMO742.

SMO was reported by myriad research as a factor in regulating cancer activities, such as colorectal cancer, glioma, *etc.* [20, 31]. Further, it was mentioned that in the liver cancer, SMO was a direct targeting of miR-338-3p. Overexpressing SMO in liver cells lines, can promote cells invasion and metastasis [32]. Same results were shown in our study. Nevertheless, what’s different in our study from the prior one is that, we uncovered the mechanism in a broader version, which involved in the role of circSMO742.

Even if we took every aspect into consideration when designed the research, we had to admit that there were some factors remained to be advanced. For instance, the number of clinical samples was simply too small to draw scientific conclusion. Ten DEGs sharing same name by differently expressed circRNA and mRNAs were screened out. And we only picked up the only up-regulated one for research. The further research should focus on investigating other down-regulated DEGs. Moreover, the development and progress of tumor was so complicated that could not cause by only several genes, such as protein-protein interaction and different signal pathways network, which should be further explored. Furthermore, the combined using of sh-circSMO742 and SMO antagonist, such as Sonidegib, Cyclopamine, SNAT-1, and MK-4101 on the treatment of glioma could be further investigated. Even so, the presented research revealed a possible pathogenesis mechanism of glioma and provided a novel target in the treatment of glioma.

## MATERIALS AND METHODS

### Glioma samples collection

10 human gliomas tissues and non-gliomas tissues (3 adjacent tissues and 7 normal brain tissue died of traffic accident) were obtained with the written informed agreement from these patients who undergoing tumorectomy in Union Hospital, Tongji Medical College, Huazhong University of Science and Technology. According to World Health Organization 2016 brain tumor classification, all the samples were identified as glioblastoma multiforme (GBM), IDH (Isocitrate dehydrogenas) wild type except one was GBM, IDH mutant type. All the samples were frozen and stored in liquid nitrogen. The process was approved and followed the ethical rule at Union Hospital, Tongji Medical College, Huazhong University of Science and Technology.

### Tissues and cells lines

Human normal nerve cell line HA, SVGP-12, cell lines from human glioblastoma A172 and U-87 MG were collected from BeNa Culture Collection (BNCC, Beijing, China). Cells were characterized by cytogenetic karyotyping and short tandem repeat (STR) profiling and passed the detection of Mycoplasma using LookOut® Mycoplasma PCR Detection Kit (Sigma-Aldrich, St. Louis, MO, USA). Cells were cultured in Dulbecco’s modified Eagle’s medium (DMEM; Solarbio, Peking, China) supplemented with 10% fetal bovine serum (GIBCO BRL, Grand Island, USA). All the cells were kept in a 37°C incubator, with a moist atmosphere and 5% CO_2_.

### Bioinformatic analysis

GSE86202 was a microarray including total RNA from 3 glioma and paired normal brain tissue. This study presented 28 common differentiated circRNAs searched by four mehods, CIRCexplorer, CIRI, find_circ and circRNA_finder, under the condition of |Fold change| > 1.5 and *P*.value < 0.05 by microarray analysis. Circular RNAs coding was transformed into 7-digital NO. and parent gene was named according to the circBase. The expressed mRNA genes analyzed with R limma and screened 2185 differentiated genes with the limitation of |Fold change| > 2 and adjusted. *P*. value < 0.05. Application of Cytoscape was aimed to build network of mRNA and circRNA and possibly targeted miRNAs transcripted from the SMO gene.

### Fluorescence in situ hybridization (FISH)

The FISH assay was performed according to the manufacture's protocol (Sino Biological Inc., Beijing, China). Briefly, after the fixed slides being dehydrated, the probes specific to circSMO742 and miR-338-3p were added to the slides, and then pre-denatured at 78°C for 5 min. Hybridization was then carried out at 42°C overnight. Nuclei were counterstained with 4,6-diamidino-2-phenylindole (DAPI, Sigma). Images were examined with a Zeiss LSM 700 Meta confocal microscope (Jena, Germany).

### QRT-PCR

RNA of glioma cells or tissues grinded into debris was extracted with Trizol reagent following the product instruction (Invitrogen, Gaithersburg, MD, USA). RNase R was utilized to confirm the stability of circSMO742 and eliminate SMO mRNA. Stem-loop-specific primer method was applied to measure expression levels of miR-338-3p. U6 was adopted as control. QRT-PCR was performed via the SYBR Select Master Mix in an ABI Prism 7000 Sequence Detection. To examine the SMO mRNA levels, reverse transcription for total RNAs was carried out by oligodT primer using RT Reagent Kit (Vazyme, Nanjing, China). The relative expression levels were evaluated with relative quantification (2^−ΔΔCt^). The primer sequences were designed according to the data deposition in public repository and shown in Table 2. SMO, NCBI, NM_005631.4; miR-338-3p, miRBase, MIMAT0000763; circSMO742, circBase, hsa_circ_0001742.

**Table 2:** Primers used in the study.

**Primers**	**Direction**	**Sequence**
SMO	F	5′-AAGACAACTTGGATTGCGAGG-3′
	R	5′-TGGGCATGTATACGGCACAC-3′
miR-338-3p	F	5′-GCAGTCCAGCATCAGTGA-3′
	R	5′-GTCCAGTTTTTTTTTTTTTTTCAACA-3′
circSMO742	F	5′-GGTGGATGGGGACTCTGTGA-3′
	R	5′-TCTTGGGGTTGTCTGTCCGA-3′
GAPDH	F	5′-CCACCCATGGCAAATTCCATGGCA-3′
	R	5′-TCTAGACGGCAGGTCAGGTCCACC-3′
U6	F	5′-CTCGCTTCGGCAGCACA-3′
	R	5′-AACGCTTCACGAATTTGCGT-3′

### Western blot

Incubated, harvested, washed with PBS buffer, cells were lysed in RIPA lysis buffer and centrifuged at 11,000 rpm, 4°C for 15 min. Supernatants were collected and loading into SDS-PAGE following by a transmembrane PVDF. After blocking with 5% skim milk, antibodies against SMO and GAPDH were diluted and added into the container. Both GAPDH rabbit mAb (#5174, 1:1000) and anti-rabbit IgG, HRP-linked secondary antibody (#7074, 1:10000) were obtained from Cell Signaling Technology (Danvers, MA, USA). And rabbit anti-Smoothened antibody (ab113438, 1:1000) was obtained from Abcam (Cambridge, MA, USA). After blot scanning, Image J software (National Institutes of Health, USA) was subsequently used for densitometric analysis of autoradiographic bands.

### Cells transfection

Vector of pLO-ciR (Geneseed) was used to overexpress circSMO742 (hsa_circ_00001742). Short interfering RNAs for circSMO742 and SMO, miR-338-3p mimics and inhibitors were all synthesized and bought from Ribobio (Ribobio, Guangzhou, China). The siRNAs for knocking down circSMO742 were transformed into shRNAs and stably cloned into lentivirus vector (HanBio, Shanghai, China) for animal experiments. Lipofectamine 2000 (Invitrogen Life Technologies) was conducted following the manufacturer's instructions.

### Biotinylated Micro-RNA pull down assay

The biotinylated miR-338-3p or mutant miR-control (constructed by replacing the predictive targeting sequences, Genechem, Shanghai, China) was transfected into U-87 MG cells. Then, the cells were fixed with 1% formaldehyde 40 h later (Sigma) at room temperature for 15 min. Application of 0.2 m glycine was to stop the reaction. Washed with TBS, the cells were lysed with lysis buffer (50 mm HEPES, pH 7.5, 140 mm NaCl, 1 mm EDTA, 1% Triton, 0.1% sodium deoxycholate), and genomic DNA was digested with RNase-free DNase I (NEB). At first, streptavidin beads (Dynabeads M-280 Streptavidin) pulled down the complex of miRNA-mRNA or miRNA-circRNA. Thereafter, the complex was washed three times with washing buffer (10 mm Tris-HCl, pH 7.5, 1 mm EDTA, 0.15 mm LiCl), and eluted with elution buffer (0.1 m NaHCO3, 1% SDS). Then a heat treatment was conducted for 10 min at 85°C. The proteins were digested as protease K added, and incubated at 65°C for 2 h to fully reverse the cross-linkages. RNA was purified with TRIzol, and the RNA level of circSMO742 and SMO were measured with qRT-PCR.

### Dual luciferase reporter assays

The SMO and circSMO742 containing the predicted miR-338 binding sites were amplified. PCR method was for the construction of Wild type (WT) plasmids while site-directed mutagenesis was used to generate mutant type (MUT) plasmids, replacing the first six ribonucleotides of the miR-338 complementary sequence. 1 × 10^5^ U-87 MG cells. Co-transfection of U-87 MG cells with plasmids and miR-338 mimics utilized the Lipofectamine 2000 transfection system (Invitrogen, Carlsbad, CA) following the manufacturer’s instruction. After the indicated reagents were treated, cells were lysed in a reporter lysis buffer (Promega, Madison, WI, USA). The firefly luciferase activities were measured with the help of the Dual-Glo Luciferase Assay System (Promega) in a single channel luminometer. Repeat all experiments in triplicate.

### Cell viability detection

After being transfected with 5 pmol scrambles or/and 1 ng vectors, cells (5×10^3^) were transferred to 96-well plates, incubating at 37°C. Each well of a 96-well plate was added with 10 μL CCK-8 reagent (Beyotime Institute of Biotechnology, Haimen, China) for 30 min at 37°C, after 0, 24, 48 and 72 h respectively. And the absorbance values (OD_490_) were read.

### Flow cytometry

Cells were planted in a 6-well plate (1×10^6^ cells/well) and kept in an incubator with ~80% confluent monolayer formed by constant 5% CO_2_ at 37°C. Then cells were transfected with 200 pmol scrambles or/and 50 ng vectors. After transfection for 48 h, cells were picked up and cleaned twice using cold PBS. Then, the cells were suspended in 100 μL 1× binding buffer (Annexin V-APC/PI Apoptosis Detection Kit, Elabscience Biotechnology Co.,Ltd, Wuhan, China) to create a single-cell suspension. Cell suspension (50 μL), 5 μL Annexin V-APC and 5 μL propidium iodide were mixed and then incubated in the room temperature for 15 min in the dark after adding 400 μL binding buffer. A flow cytometer (EPICS, Xl-4; Beckman Coulter, Inc., Brea, CA, USA) was used to detect the samples. The software used for data analysis was FlowJo v 10 (TreeStar, Inc., Ashland, OR, USA).

### Evaluation of cell mobility

The mobility of glioma cells was determined by Transwell chamber inserts (BD Biosciences, Franklin Lakes, NJ, USA) with or without Matrigel (for invasion assays, D Biosciences, San Jose, CA, USA). 5×10^3^ cells were plated in the upper chamber without serum. Medium mixed with 10% FBS was added to the lower chamber of the inserts. U-87 MG cells were incubated at 37°C for 36 h for the migration assay, and 48h for invasion assay. Non-migrated cells were totally removed by a cotton swab. Cells migrated or invaded to the other side of the membrane were stained with 0.1% crystal violet for 25 min at 25°C. Cell number was counted for in three fields of view using a microscope (Leica DMIRB Inverted Fluorescence Microscope, Leica Microsystems GmbH, Wetzlar, Germany).

### Tumor xenograft

BALB/c female nude mice aging six to eight weeks, were used in the experimental procedures. U-87 MG cells with circSMO742 knocking down, stably transfected with sh-Circs, were subcutaneously and intracranially implanted into nude mice, respectively. Cells were harvested and re-suspended in PBS. For the detection of tumor size and weight as well as the expression of relative RNAs and proteins, 18 mice were randomly and equally divided into 3 groups, NC, sh-circ-1, sh-circ-2. 5×10^6^ cell suspensions were subcutaneously inoculated in the flank of nude mice. The tumor size and weight were separately calculated during 20 days and the tumor weight was measured after removing excess water. Mice were sacrificed to measure tumors on 20^th^ day. Expression levels of Circ, miR-338-3p and SMO were then detected after that. For orthotopic inoculations, another 18 mice were randomly and equally divided into 3 groups, NC, sh-circ-1, sh-circ-2, and intracranially injected with 1×10^7^ transfected U-87 MG cells. The number of survived nude mice was registered and survival analysis was performed using Kaplan-Meier survival curve. This study had gained approval of the ethics committee of Union Hospital, Tongji Medical College, Huazhong University of Science and Technology.

### Histochemistry

The tumor tissue samples were dehydrated and paraffined following the routine methods. Sections were cut by different operators blinded to aim. Then, paraffin were removed. Paraffin section were rinsed in PBS-T for 3-5 minutes and were blocked with 3% peroxide-methanol for endogenous peroxidase ablation. Discs were incubated with anti-Ki67 antibody (1:200, Cell Signaling Technologies, Danvers, MA) overnight at 4°C, after being blocked with PBS, 1% BSA and 0.3% triton X-100 for 1 hour. Washed four times in blocking buffer, discs were maintained with the secondary antibody for 1 hour at room temperature in the dark. Images were screened with a LSM710 (Zeiss) confocal microscope. Adobe Photoshop subsequently processed the photos.

### Statistical analysis

All the experiments were performed at least three times. The results was shown as mean ± SD. Normality test and paired Student's t-tests was applied to analyze the results of assays. One-way analysis of variance (ANOVA) was first used to assess whether a difference was existing in two or more group, subsequently, using tukey’s multiple comparison test to assess for a significant difference among the individual groups. All statistical calculations were performed using GraphPad Prism 6.0 software (La Jolla, CA, USA). A significant statistics difference was determined with a P value less than 0.05.

### Ethical approval

All procedures performed in studies involving human and animal participant were in accordance with the ethical standards of the Union Hospital, Tongji Medical College, Huazhong University of Science and Technology.

## Supplementary Material

Supplementary Figure 1
