# Supplementary material for: Circular RNA SMO sponges miR-338-3p to promote the growth of glioma by enhancing the expression of SMO
Source: Aging (Albany NY). 2019 Dec 30;11(24):12345–60. doi: 10.18632/aging.102576 (PMC6949074; doi:10.18632/aging.102576)
Supplement: Supplementary Figure 1 [file aging-11-102576-s001..pdf]

SUPPLEMENTARY FIGURE

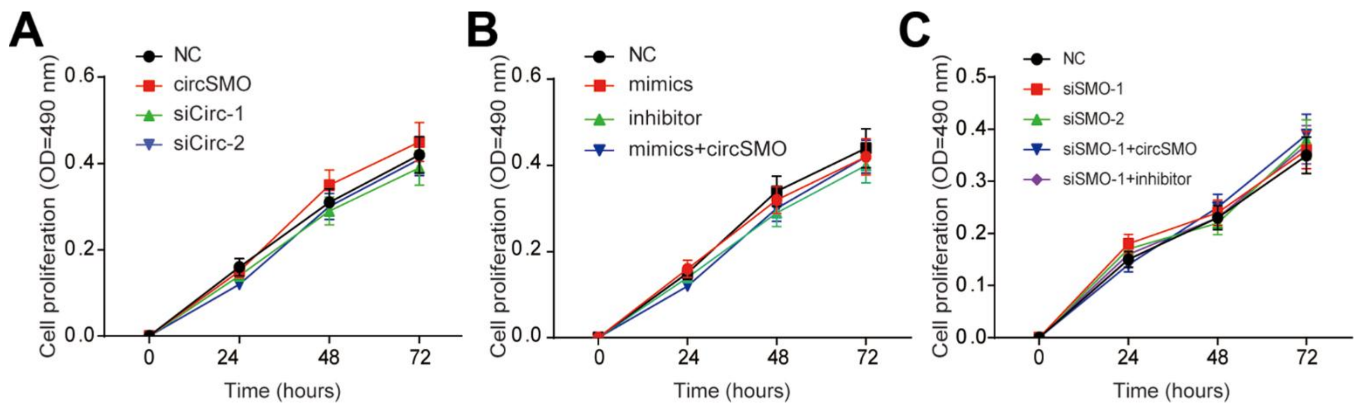

Supplementary Figure 1. Transfection of circSMO742, miR-338-3p, and SMO had no effects on the HA cell line.
